# Accelerated clearing and molecular labeling of biological tissues using magnetohydrodynamic force

**DOI:** 10.1038/s41598-021-95692-2

**Published:** 2021-08-12

**Authors:** Joseph Dwyer, M. Desmond Ramirez, Paul S. Katz, Rolf O. Karlstrom, Joseph Bergan

**Affiliations:** 1 Neuroscience and Behavior Graduate Program, University of Massachusetts Amherst, Amherst, USA; 2 Department of Biology, University of Massachusetts Amherst, Amherst, USA; 3 Department of Psychological and Brain Sciences, University of Massachusetts Amherst, Amherst, USA

**Keywords:** Histology, 3-D reconstruction, Fluorescence imaging

## Abstract

Techniques used to clear biological tissue for fluorescence microscopy are essential to connect anatomical principles at levels ranging from subcellular to the whole animal. Here we report a simple and straightforward approach to efficiently render opaque tissue samples transparent and show that this approach can be modified to rapidly label intact tissue samples with antibodies for large volume fluorescence microscopy. This strategy applies a magnetohydrodynamic (MHD) force to accelerate the removal of lipids from tissue samples at least as large as an intact adult mouse brain. We also show that MHD force can be used to accelerate antibody penetration into tissue samples. This strategy complements a growing array of tools that enable high-resolution 3-dimensional anatomical analyses in intact tissues using fluorescence microscopy. MHD-accelerated clearing is simple, fast, reliable, inexpensive, provides good thermal regulation, and is compatible with existing strategies for high-quality fluorescence microscopy of intact tissues.

## Introduction

Advances in microscopy now allow investigation of subcellular anatomical structures while maintaining the macroscopic organization of intact tissues. Generating high-quality tissue samples is a critically important step towards achieving this goal. Most biological tissues, including the brain, are recalcitrant to large-volume microscopy without first being made optically transparent (cleared). Early methods for chemically based tissue clearing quenched fluorescence, making tissue samples unsuitable for fluorescence microscopy^1,2^; however, modern approaches for tissue preparation reduce light scattering without quenching fluorescence^3–8^ (Table 1). These approaches reduce light scattering primarily by removing lipids and standardizing the refractive index of the tissue sample. When combined with genetically encoded fluorophores, these approaches enable anatomical investigation with sub-micron precision at depths of at least a centimeter. Here, we present a technique that utilizes MHD force in combination with a conductive buffer and detergent to efficiently, reliably, and cost-effectively prepare high-quality cleared tissue samples for visualization with fluorescence microscopy. Importantly, MHD-based clearing minimizes thermal damage to tissue, preserves endogenous fluorescent signals, and is simple to implement.

**Table 1:** A direct comparison of multiple popular clearing techniques, based on literature, that shows the reported time it takes to clear an intact mouse brain, the relative antibody penetration into the tissue over a single hour, the degree of difficulty to setup and use the technique, and the amount of money it costs to implement the technique effectively.

Technique	Time to clear full mouse brain (h)	Antibody penetration over time (mm/h)	Level of difficulty	Cost
MHD-accelerated clearing	12–48	0.15	Low	$
CLARITY^3^	120–216	0.0074	High	$$
Stochastic electrotransport^5^	72	0.20	Very high	$$
ACT-PRESTO^9^	6	0.040	High	$$$
SCALEs^10^	72	0.066	Medium	$
uDISCO^11^	198	0.0046–0.010	Low	$
CUBIC/CUBIC-HistoVision^12,8^	72–168	0.007–0.060	Medium	$
Adipo-Clear^13^	24–48	0.042–0.050	Medium	$
SWITHCH^14^	168–672	0.083	Low	$

Degree of difficulty is a subjective measure of the amount and complexity of steps and solutions required to implement each technique and the level of expertise required construct devices for required for the technique and use these devices to clear mouse tissue. Level of difficulty ranges from easy (easy setup and/or requiring very few easy steps) to very hard (intricate setup that requires a high level of specialized expertise and/or requires many difficult steps) Cost to implement the technique is displayed as less than $1000 ($), less than $10,000 ($$), and over $10,000 ($$$).

**Figure 1:**
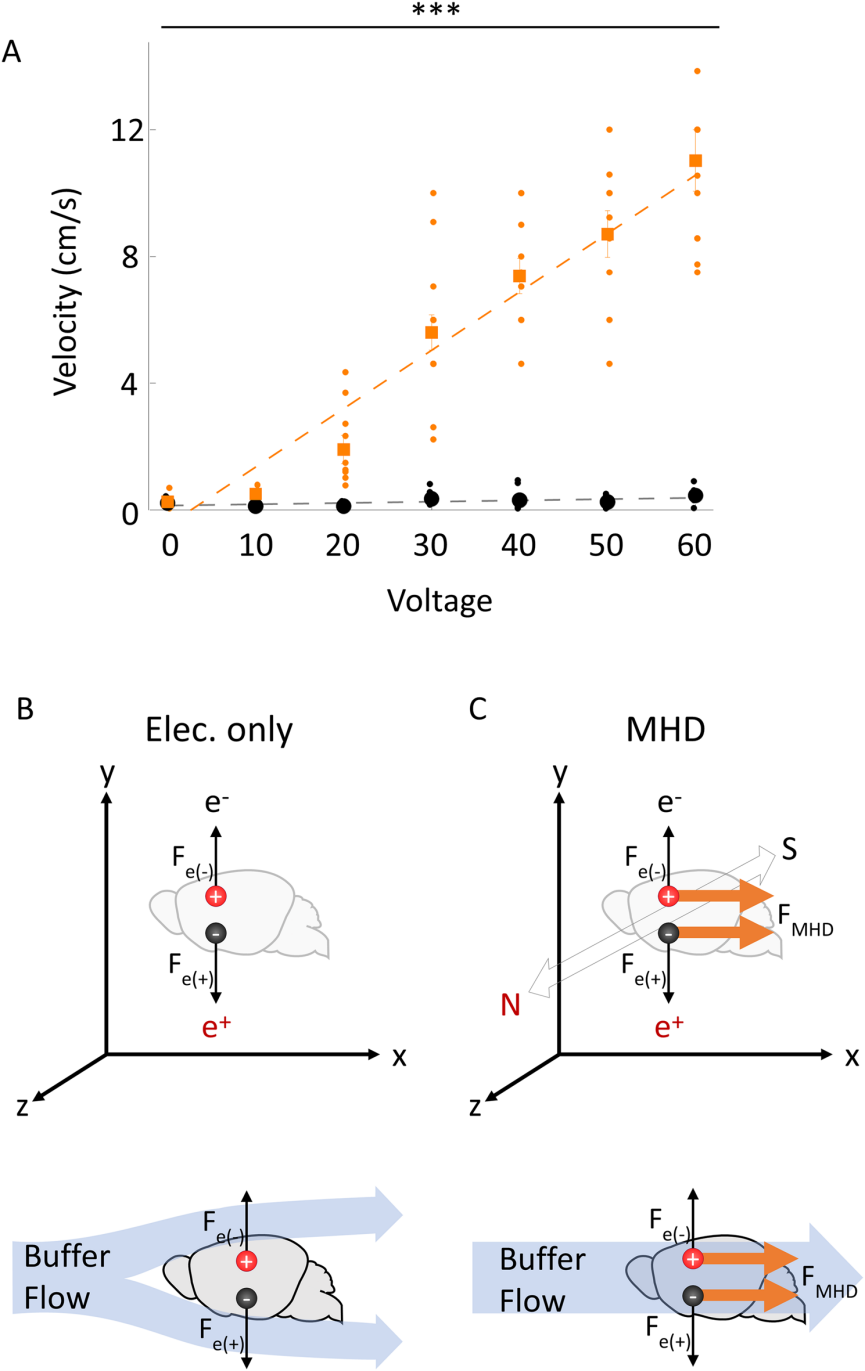
Comparison of voltage effects on buffer velocity between MHD and electrical forces. (**A**) Velocity of sodium alginate spheres through the MHD-accelerated clearing device with (orange) and without a magnetic field (black; N = 7; error bars: standard error of the mean). MHD-acceleration increases produced higher velocities than electric-only across all voltages (*p* < 0.0001 (***); 2-way ANOVA (F{2,6} = 38.51; pairwise comparisons: 10 V: *p* = 0.002, 20 V: *p* = 0.0005, 30 V: *p* < 0.0001, 40 V: *p* < 0.0001, 50 V: *p* < 0.0001, 60 V: *p* < 0.0001). The MHD and electric datasets are fit to linear models *y* = 0.18*x* − 0.48 (orange) and *y* = 0.004*x* + 0.15 (grey) respectively. Illustrations of the effects of an electric field (**B**) or conjugated electric and magnetic field (**C**) on positively charged (red) or negatively charged (black) particles. The force induced on each particle by the electric field alone (black arrow) and MHD force (orange arrow) are shown as vectors. The cartoons below show the buffer flow induced by an external pump in conjunction with electrical force (**B**) or by MHD force alone (**C**). The flow of the buffer is shown as blue arrows where the MHD force (orange vector) continues to push buffer through the tissue, while the external pump produces flow around the tissue.

MHD force describes a physical phenomenon also known as Lorentz force where force is generated on a charged particle in the third orthogonal direction from perpendicular electric and magnetic fields^15^. The efficiency of MHD force to rapidly drive charged molecules into and out of tissue is a consequence of a fundamental difference in the way that MHD fields and electrical fields act on charged particles. Electrophoresis drives cations and anions in opposite directions resulting in no net flow of buffer through a tissue sample. In contrast, MHD-forces drive cations and anions in the same direction along the third orthogonal axis resulting in a unidirectional flow of buffer through the sample itself (Fig. 1C^15^). The rapid flow of buffer through a tissue sample located within the MHD field (Video 1) constantly replaces heated buffer with fresh cool buffer thereby minimizing thermal damage to fluorescent molecules embedded in a large tissue sample while rapidly removing unbound molecules.

Intact tissues also present a challenge for the introduction of molecules that are needed to label molecular features deep in the sample. Based on the efficacy of MHD-accelerated clearing, we tested if MHD force could be used to propel antibodies into tissue samples. The same approach used to remove lipids and clear tissue samples also accelerated the penetration of antibodies into the tissue sample—MHD-accelerated labeling.

Using MHD-accelerated clearing, transparency of an intact mouse brain can be achieved in as little as 12 h. MHD forces can subsequently be harnessed to drive antibodies into cleared tissues. These MHD-based approaches work in both vertebrate (shown for mouse and zebrafish) and invertebrate (shown for the nudibranch mollusk *Berghia stephanieae*) species, providing a generalizable method to render intact tissue transparent and accelerate immunohistochemical labeling for fluorescence microscopy of intact tissues. We provide plans for the construction of the MHD device, as well as a detailed protocol to ensure the successful implementation of this strategy for those interested in large-volume tissue microscopy.

## Results

### Effects of MHD force

MHD force produces a linear increase in flow velocity that is not observed with the application of purely electrical force (Fig. 1). To quantify the effects of the MHD force, we compared the movement of sodium alginate spheres suspended in an electrically conductive buffer in response to purely electrical or MHD forces. The MHD condition produced dramatically higher velocity flow over the electrical only condition for all tested non-zero voltages and across the time course (Fig. 1A; *p* < 0.0001). The difference between MHD and electric-only flow velocity increased as the applied voltage increased (Fig. 1A).

### Tissue clearing/delipidation

**Figure 2:**
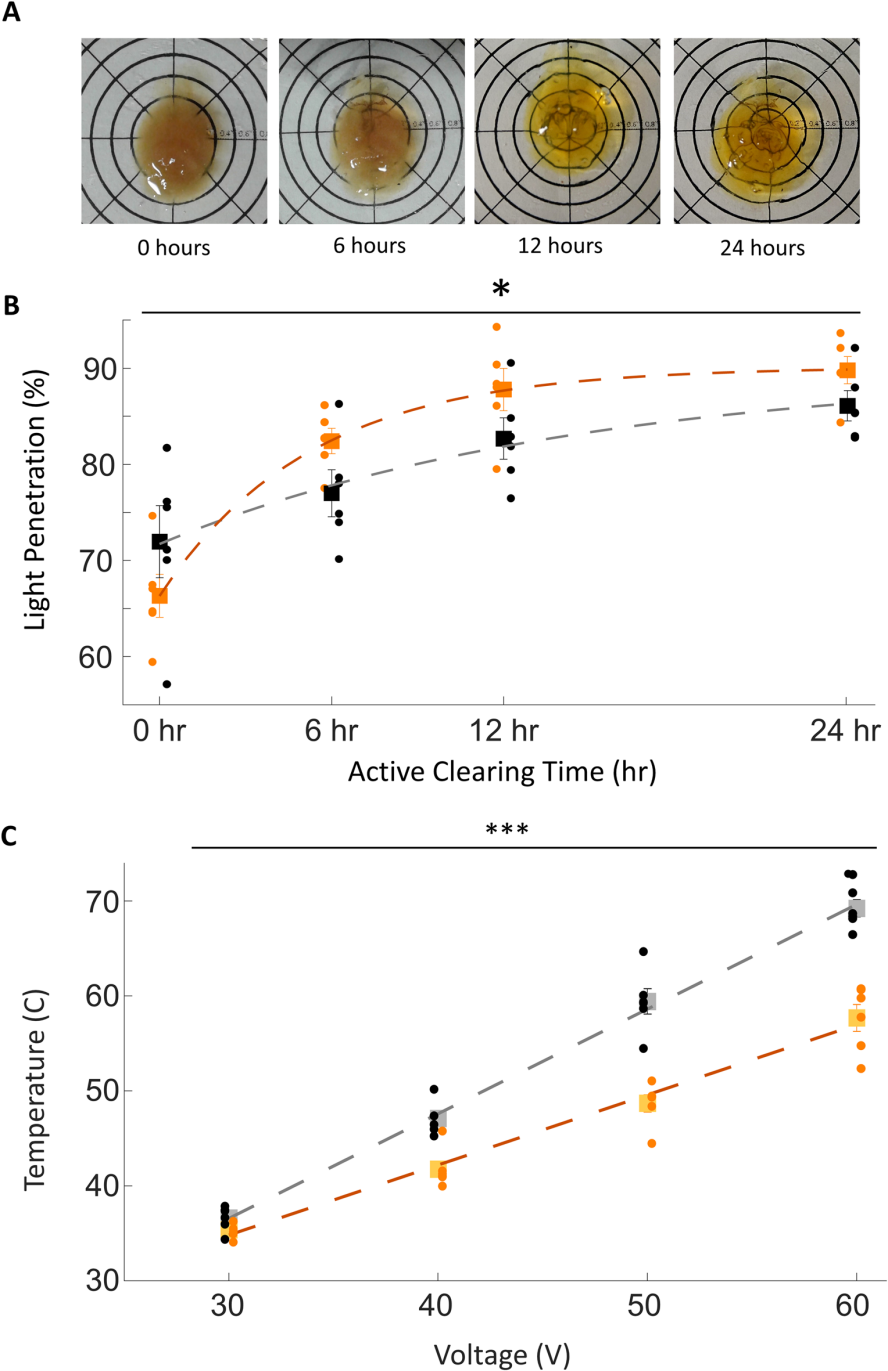
MHD-accelerated clearing of the intact mouse brain. (**A**) Representative examples of intact cleared brains actively cleared with MHD for 0, 6, 12, and 24 h and then equilibrated in RI-matching solution (N = 6). (**B**) Measurements of the optical transparency of mouse brains cleared using MHD force (red) or electrical force combined with a pump to circulate buffer solution (black). Transparency was measured as percentage wide-spectrum light penetration through the tissue. Both MHD and electric only data were fit to saturating exponentials (MHD: $y = 89.9 - 18.2*e^{ - 0.07x}$; Electric-only: $y = 90.1 - 23.8*e^{ - 0.19x}$). A significant interaction between electrical and MHD clearing over time is denoted by an asterisk F{2,3} = 3.24, *p* = 0.03. (**C**) Average peak temperature of tissue actively cleared with MHD (orange) was lower than electric-only (black) at voltages ranging from 30 to 60 V (two-way ANOVA F{1,3} = 119, *p* < 0.0001; linear fit for electric-only $y = 1.1x + 3.6$; linear fit for MHD: $y = 0.7x + 12.7$.

MHD-accelerated clearing renders an intact mouse brain transparent in as little as 12 h (Fig. 2). Both electric-only and MHD-accelerated clearing remove lipids from brain tissue and produce an increasingly transparent tissue sample with longer clearing times (Fig. 2B). High-quality images can be produced with both Electric-only and MHD-accelerated clearing, and no differences in shape were observed for tissue samples prepared with MHD-accelerated versus electric-only clearing (length, width, volume; *p* > 0.4; N = 5; Fig. S1); however, samples prepared with MHD-accelerated clearing (30 VDC; 0.35 AMPS) were ready to be imaged in less time than electric-only clearing (30 VDC; 0.35 AMPS) and exhibited better imaging (Fig. S2). As the electric-only condition does not produce buffer flow independently, these trials were conducted with the assistance of a peristaltic pump (500 ml/min) with flow matched to the MHD condition to prevent tissue damage from overheating.

Because excessive heating during active clearing can denature proteins and quench fluorescence, we measured the temperature of tissue samples actively cleared with either MHD or electric-only conditions at matched voltage, amperage, and buffer circulation (Fig. 2C). Note that the buffer circulation for the MHD condition is intrinsic to the technique while a pump is required to achieve buffer flow with the electric-only condition. The temperature of brains cleared in the electric-only condition were higher than those cleared in the MHD condition across the full range of tested voltages indicating that MHD-accelerated clearing provides additional thermal buffering (F{1,3} = 119, *p* < 0.0001; 2-way ANOVA with repeated measures).

**Figure 3:**
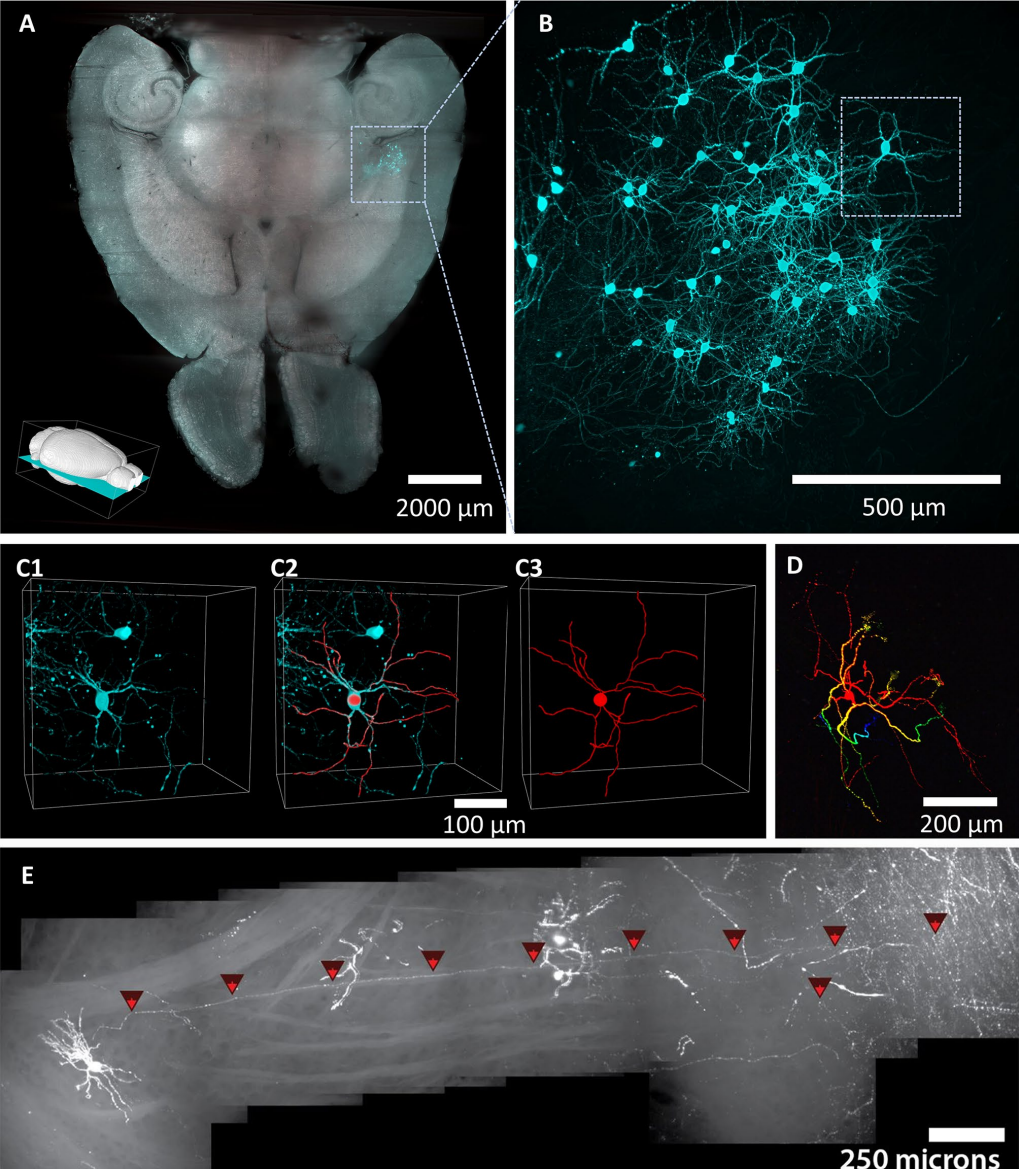
Light sheet microscopy with MHD-cleared tissue. (**A**) Optical slice of an intact mouse brain cleared using MHD-accelerated clearing (horizontal orientation; inset: position of slice). GFP-labeled cells (cyan) are clear in the medial amygdala. (**B**) Higher magnification image corresponding to the location of the dashed box in panel A showing individual cells and associated neural processes are visible deep within the tissue. (**C**) Higher magnification corresponding to the dashed box in panel B showing an isolated GFP-expressing neuron in the MeA (left), traced neural processes (right), and overlay of the fluorescent image and trace (center). (**D**) A depth color-coded projection of a single GFP-expressing neuron cell inside the brain (100 µm depth for projection; red: closest; blue: farthest). (**E**) Single axon traced from cell body to axon terminals through several millimeters of brain tissue inside the intact brain. The cells shown in (**D**) and (**E**) are not from the same sample as in (**A**) through (**C**).

MHD-accelerated clearing reliably rendered tissue samples optically transparent while also preserving genetically encoded fluorescent proteins (Fig. 3). An intact adult mouse brain conditionally expressing GFP via EnvA-G-deleted rabies virus in cells that project to the aromatase-expressing neurons in the medial amygdala^16–18^ was prepared using MHD-accelerated clearing. Without fluorescent labeling, the shadows of individual cells and fine subcortical architecture (e.g., anterior commissure) is visible into the very center of the tissue (Fig. 3A). A population of GFP-expressing cells was easily identified in the medial amygdala (Fig. 3A). Higher magnification images showed that fine processes, such as dendrites and axons, can be identified and analyzed several millimeters (3 mm) from the surface of the brain (Fig. 3B; Video 2). Indeed, the resolution is sufficient to reconstruct the dendritic arbors of individual neurons in three dimensions (Fig. 3C,D) and to reconstruct the path of a single axon, including axon collaterals, from the cell body, through several millimeters of brain tissue, ending at the axon terminals (Fig. 3E).

### Tissue labeling

**Figure 4:**
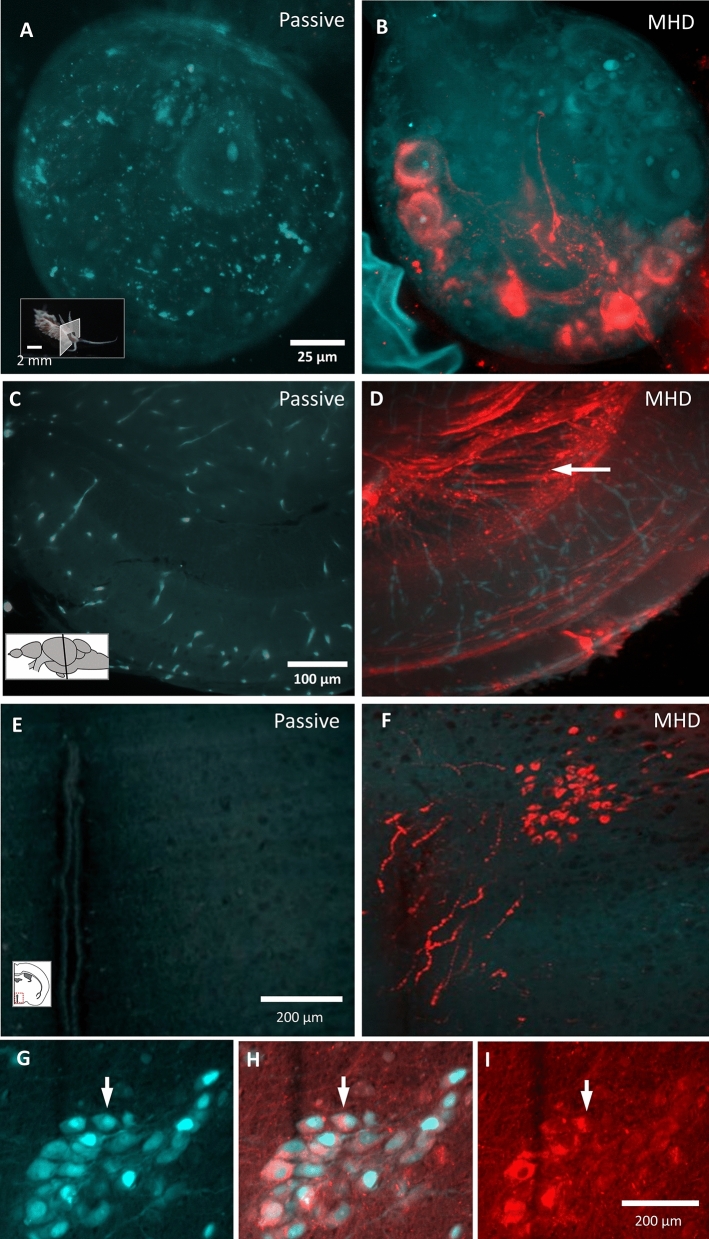
MHD-accelerated antibody labeling of brain tissue from sea slug, zebrafish, and mouse. (**A**, **B**) Image of an intact Berghia stephanieae pedal ganglion after passive (A) and MHD-accelerated (**B**) α-serotonin antibody labeling (red) with tissue autofluorescence (cyan). (**C**, **D**) Images of a cleared adult zebrafish brain (3 mm x 3 mm x 6 mm) after α-acetylated tubulin antibody labeling (red) with tissue autofluorescence (cyan; Passive labeling: C; MHD-accelerated labeling: D). (**E**, **F**) Images of cleared mouse brain sample (6 mm x 6 mm x 6 mm) after α-oxytocin labeling (red) with tissue autofluorescence (cyan; Passive labeling: E; MHD-accelerated labeling: F). (**G**–**I**) MHD-accelerated labeling of adult mouse brain sample (6 mm x 6 mm x 6 mm) after α-vasopressin antibody labeling (cyan) with genetically encoded tdTomato in vasopressin-expressing neurons (red; AVP-cre X rosa26-lsl-tdTomato; Pearson’s coefficient: r = 0.609). Insets indicate the imaging plane.

MHD-accelerated labeling accelerated penetration of charged molecules and allowed labeling of large intact tissue samples (Fig. S3). To confirm the specificity of antibody binding is maintained in MHD-accelerated labeling, we used an anti-vasopressin antibody in mice that expressed tdTomato in vasopressin-expressing neurons (Fig. 4G–I). Tissue was generated by crossing the Ai9 Rosa26:LSL:tdTomato reporter line^19^ and a line where Cre recombinase is expressed under the control of the arginine vasopressin (AVP) promoter^20^. This produced tissue where the fluorescent reporter tdTomato was expressed under the control of the AVP promoter. After a 12-h MHD accelerated antibody label on this tissue using an anti-AVP antibody, we observed specific co-labeling of the genetically encoded fluorophores and the anti-AVP antibody (Pearson’s coefficient: 0.61,Fig. 4G–I).

An intact adult nudibranch (*Berghia stephanieae*) (medio-lateral: 1.3 mm, dorso-ventral: 1.5 mm, anterio-posterior: 2 cm) that had been delipidated using the MHD-accelerated clearing device was incubated with an anti-serotonin (5-HT; Immunostar; 1:500) antiserum followed by a fluorescent secondary antibody (488 nm conjugated; ThermoFisher; 1:200) suspended in a high pH electrophoresis buffer (0.1 M Borate Buffer and 0.1% Triton X-100 brought to pH 9.5 with 0.1 M LiOH; Fig. S4). Passive incubation for 12 h resulted in little to no penetration into the brain (Fig. 4A), whereas MHD-accelerated antibody labeling for 12 h drove antibodies throughout the sample and revealed 5-HT expressing cell bodies and neurites (Fig. 4B).

Intact zebrafish brains (medio-lateral: 3 mm; dorso-ventral: 3 mm; anterio-posterior: 6 mm) were passively delipidated in SDS for 7 days and then incubated with anti-acetylated tubulin antiserum (Immunostar; 1:500^21^) for 12 h to identify neural fibers (Fig. 4D). Control tissue samples (no MHD force applied) showed minimal antibody penetration along the outer edge of the tissue with little fluorescence visible in the optic tectum (Fig. 4C). In contrast, MHD-accelerated labeling for the same amount of time showed robust labeling of neural tracts throughout the brain (Fig. 4D).

To test MHD-accelerated labeling in mammalian tissue, an anti-oxytocin (OT) antibody was applied to a cube of mouse brain (medio-lateral: 6 mm, ventro-dorsal: 6 mm, antero-posterior: 6 mm) centered on the periventricular nucleus of the hypothalamus (1:500 primary; 1:200 secondary; Fig. 4F). As above, antibodies did not effectively penetrate the tissue sample in the absence of MHD force (Fig. 4E). In contrast, OT-expressing cells were clearly visible in the PVN, located deep within of tissue cube, using MHD-accelerated labeling (Fig. 4F). OT-expressing neuronal processes were easily resolved and were seen to project towards the third ventricle, which is consistent with OT neuron morphology (Fig. S4; Fig. 4G–I). Accurate OT-labeling was seen > 1.8 mm from the nearest edge. The ability to visualize axonal varicosities and nuclei in OT-labeled neurons demonstrated that the MHD-accelerated labeling strategy can be used to resolve subcellular structures (Fig. 4F). MHD-accelerated antibody labeling was also effective for labeling an intact mouse brain hemisphere and can be used for dual antibody labeling (Fig. S5).

## Discussion

The ability to study fine anatomical structures while maintaining their native organization is necessary to reveal relationships at the wide range of scales over which biological functions occur. The MHD-accelerated protocol outlined here harnesses the strengths of already valuable electric hydrogel-based clearing approaches to maintain proteins and genetically encoded fluorescence in large samples^3,5,9,11,12^. MHD accelerated clearing maintains all advantages of electric-only clearing and adds the additional MHD force to further accelerate tissue clearing without increasing the potentially damaging electric field (Table 1,Fig. 2; Fig. S1; Fig. S2).

MHD-induced flow serves at least three purposes. First, in the case of tissue clearing MHD accelerates the removal of lipids from tissue samples. Second, in the case of antibody labeling it helps push antibodies into the tissue. While MHD acts directly on electrically charged antibodies, it is also possible that the observed acceleration of antibody penetration is because MHD generates buffer flow inside the fixed tissue. We propose that antibodies and lipids may be pulled by the flow of buffer through the tissue sample allowing rapid clearing and labeling. Third, MHD driven buffer flow provides additional thermal regulation of tissue samples above that observed with electrophoretic-only approaches—guarding against tissue damage (Fig. 1). While loss of fluorescence during clearing depends on many factors (e.g., sample size, amperage, fluorophores, and temperature), excessive heating is a primary concern for active tissue clearing. For example, tissue reaches the denaturation point for GFP (70 °C^22^) at roughly 60 V when using electric-only clearing while the same voltage results in a far safer temperature (57 °C) when using MHD-accelerated clearing (Fig. 2). While MHD-accelerated and electric-only clearing approaches likely produce similar heat when the voltage and current are matched, MHD-accelerated clearing pulls fresh buffer into the tissue sample to replace buffer that has been heated by the electrical resistance—reducing the risk of tissue damage, loss of fluorescence, and protein denaturation. Because no moving parts are required to maintain MHD-accelerated buffer flow and the resultant thermal buffering, tissue damage resulting from pump failure during active clearing is almost completely eliminated with MHD-accelerated tissue clearing.

**Figure 5:**
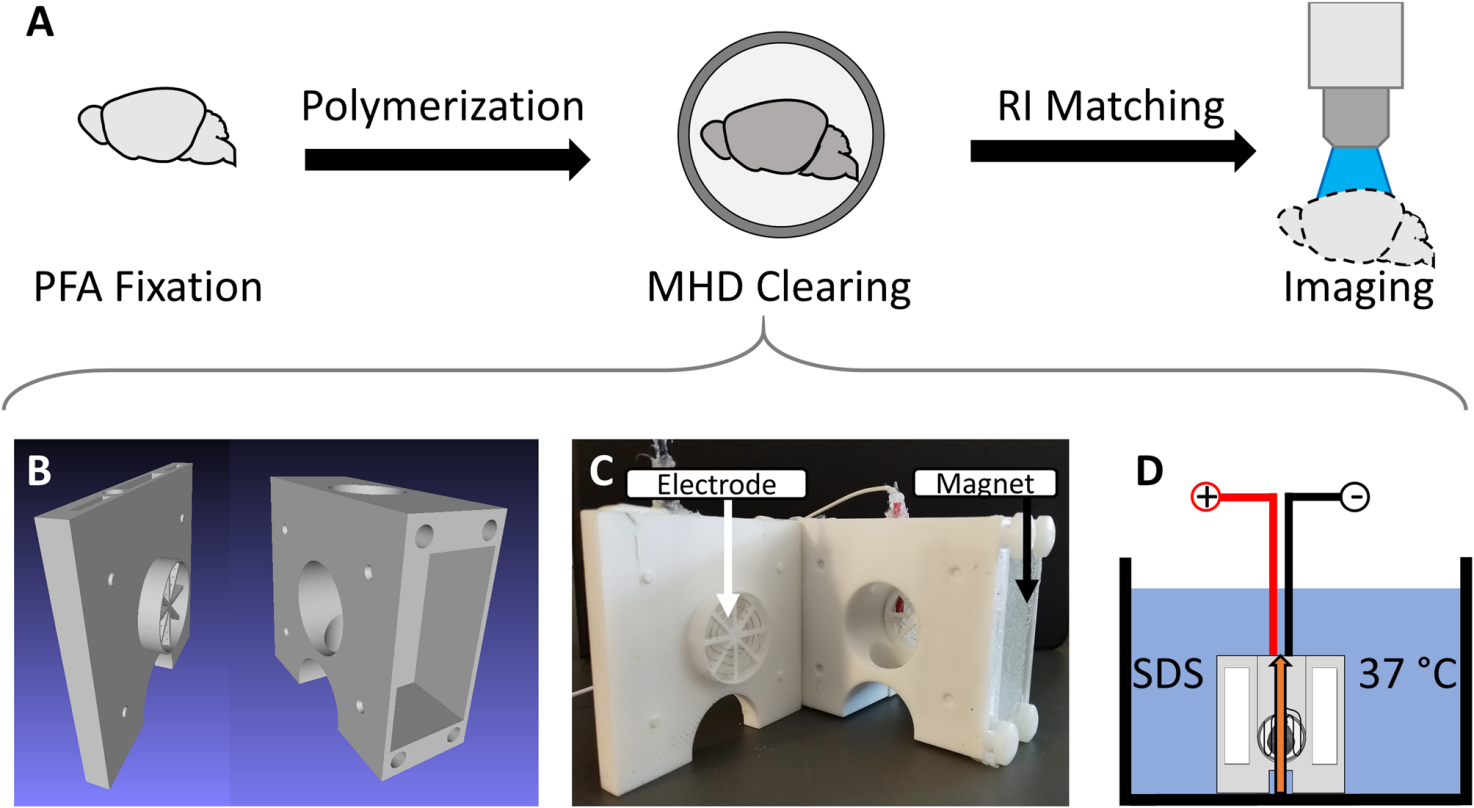
Overview of MHD-accelerated clearing approach. (**A**) Steps required to effectively clear tissue of lipids. (**B**) CAD diagram showing the MHD-assisted clearing device. (**C**) A photograph of the clearing device with tissue chamber exposed and arrows to show the location of the magnets and electrodes in the device. (**D**) Illustration of clearing device submerged in a container filled with detergent solution held at 37 °C. Tissue is placed in the central chamber where MHD force (orange arrow) produced from the electrical and magnetic fields simultaneously circulate the buffer solution and accelerate clearing.

MHD-accelerated clearing does not rely on solvents that are harmful to fluorophores (e.g., methanol and hydrogen peroxide), and simplifies tissue clearing to the bare minimum components while maintaining the core approach outlined in the original CLARITY protocol^3^. The only obligatory requirement is that the tissue sample is held at the intersection of an electrical and a magnetic field. Thus, the strategy outlined here is clean, efficient, and readily adaptable (Table 1). The device itself can be 3D printed in plastic (Fig. 5; manufacturing instructions included in Supplementary Files) making the device simple and cost-effective, roughly two-hundred dollars, to build. By speeding up the time needed to actively clear tissue and by reducing the risk of tissue damage during clearing and antibody labeling, MHD-accelerated clearing may provide benefits when extensive clearing or labeling are required (e.g., multiple rounds of antibody labeling^3^).

**Figure 6:**
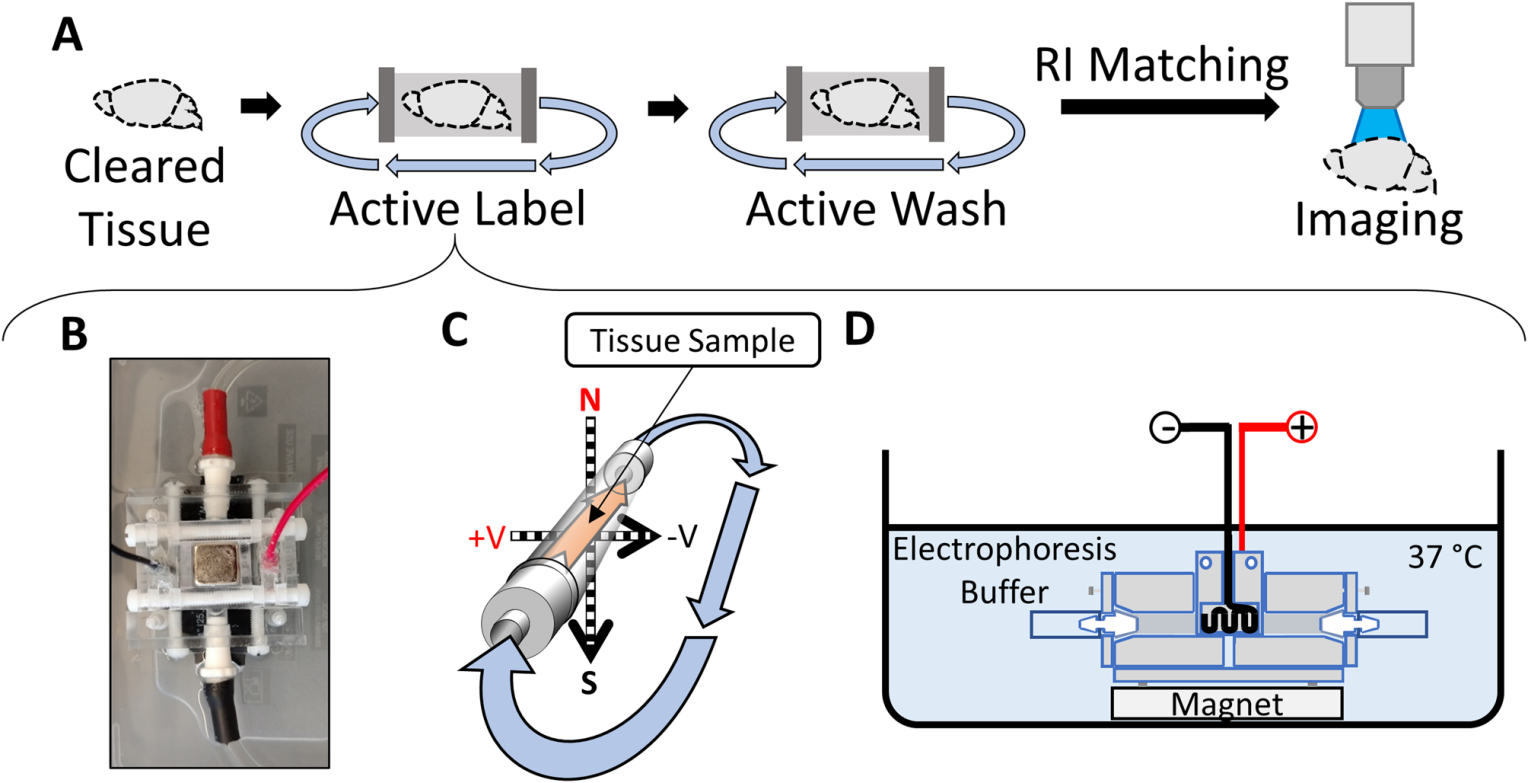
Overview of MHD-accelerated antibody labeling. (**A**) Illustration of the steps required to label and image tissue. (**B**) Picture of the MHD-assisted labeling device. (**C**) Schematic showing the tissue location inside the MHD-assisted labeling device. The direction of the MHD force is indicated by the orange arrow inside the dialysis tubing. The resulting direction of flow of the solution through the closed loop is indicated by the blue arrows. (**D**) Diagram of the antibody labeling device setup for a label. The device is submerged in a bath of electrophoresis buffer held at 37 °C.

The MHD-based approach described here (Figs. 5, 6) reliably allows rapid tissue clearing, rendering them suitable for three-dimensional fluorescent imaging. We demonstrate the efficacy of a simple MHD device by clearing dozens of mouse brains and measuring the effects of voltage and MHD-conjugation on tissue heat, clarity and time needed to achieve complete optical transparency. We show that MHD-accelerated clearing is faster and maintains a safe temperature for tissue samples over a wider range of voltages than electric-only clearing. Beyond tissue clearing, we introduce the possibility of labeling tissue samples including sea slug, zebrafish, and mouse with fluorescent antibodies. Each antibody protocol required no more than 4.5 mL (1:200 concentration) of labeling solution and both the buffers and antibody solutions can be collected at the end of the procedure and reused. Taken together, we believe MHD-accelerated clearing and labeling provides a simple, reliable, effective, and economical approach that can also be quickly adapted to the specific needs of each experiment.

## Methods

All vertebrate animals were handled according to protocols approved by the UMass Amherst Institutional Animal Care and Use Committee (IACUC; protocol #2018-0014 and #2017-0060) in accordance with the Public Health Service Policy on Humane Care and Use of Laboratory Animals. All the procedures for animal use, data collection, and data analysis adhered to the ARRIVE guidelines with the limited exception that antibody labeling and clearing effectiveness was measured unblind. In these instances of unblinded data collection, the data analysis was conducted blind.

### Measure of MHD-induced flow

A solution of sodium chloride was made in a small tank (2.5 L). Sodium chloride was slowly added to the tank until the electric conductivity of the solution matched that of the clearing solution. The clearing device was then submerged in the solution with a measured grid behind the tank to provide scale. 0 V, 10 V, 20 V, 30 V, 40 V, 50 V, or 60 V were applied to the device and sodium alginate spheres were introduced into the tank at a constant location (N = 7). The velocity of the spheres through the device was measured. Velocity was calculated using a high-speed video taken over a calibrated grid. This process was then repeated using only an electric field (magnets were removed). Paired-sample t-tests were performed between the MHD and electric-only conditions at each voltage and a 2-way ANOVA was performed across all voltages using MATLAB. The p-values for the paired samples T-test were corrected for multiple comparisons using Bonferroni correction. Each condition was fit to a linear model using MATLAB.

### Design of MHD-accelerated clearing device

The strategy for using MHD to remove lipids from tissue samples requires binding proteins and polymerizing a hydrogel, removing lipids, and matching the refractive index of the tissue and imaging media (Fig. 5A). A tissue chamber was placed into the central chamber of the MHD-accelerated clearing device (Fig. 5B,C). This holds the tissue at the intersection of the electrical and magnetic fields. The clearing chamber was submerged in a large (5 L) bath of clearing solution at 37 °C and 30 VDC (0.35 Amps) was applied across the tissue for several hours (typically 16 h for mouse brain tissue and 2 h for intact zebrafish brains; Fig. 5D).

### Tissue fixation and hydrogel polymerization

Mice were anesthetized with isoflurane, euthanized, and perfused with 0.01 M phosphate buffered saline (PBS) followed by 4% paraformaldehyde (PFA) in 0.01 M PBS. Tissue was then post-fixed in 4% PFA at 4 °C overnight. Next, the tissue was placed in a hydrogel solution (4% acrylamide, 4% PFA, 0.05% bis acrylamide, and 0.25% VA-044 initiator suspended in 0.01 M PBS) at 4 °C overnight^3,23^. Oxygen was flushed out of hydrogel-infused tissues nitrogen gas and then the samples were polymerized by incubating them at 37 °C overnight^3^. Excess hydrogel was removed from the surface and tissue samples were transferred to PBS to flush hydrogel monomers.

Adult zebrafish were euthanized in 0.2 mg/ml tricaine mesylate (MS-222), decapitated, and the heads placed in 4% paraformaldehyde overnight. Heads were then placed in PBS and brains were carefully dissected, incubated in hydrogel at 4 °C overnight, and processed as above.

Adult nudibranchs (*Berghia stephanieae*) were anaesthetized in cold 4.5% magnesium chloride in artificial sea water for 20 min, pinned to a Sylgard-lined dish, and fixed in 4% paraformaldehyde in sea water overnight at 4 °C. Whole animals were washed with PBS and then incubated in hydrogel at 4 °C overnight and processed as above.

### Active tissue delipidation (clearing)

Tissue samples were incubated in SDS-clearing solution (10 mM sodium dodecyl sulfate in 0.1 M borate buffer, pH 8.5) for 2 days at 37 °C unless otherwise noted. Samples were then transferred to the MHD-accelerated clearing chamber, consisting of two interlocking cell-strainers (ThermoFisher; catalog #: 87,791). This chamber was placed in the intersection of the electrical and magnetic fields in the center of the device and the chamber was lowered into a bath of 37 °C SDS. 30 V DC were then applied across the tissue to initiate MHD-accelerated clearing (Fig. 5D). After clearing, the tissue is taken out of the clearing chamber and washed in 0.1 M PBS for at least 12 h. Of the 55 samples cleared for this paper using this technique in multiple laboratories, all achieved transparency with little physical damage.

### Electrophorectic clearing

Tissue samples were incubated in SDS-clearing solution for 2 days at 37 °C unless otherwise noted. Samples were then transferred to a clearing chamber, consisting of two interlocking cell-strainers (ThermoFisher; catalog #: 87,791). This chamber was placed between two electrodes in the center of an MHD-accelerated clearing device, which has had magnets removed from the device. A 500 ml/min peristaltic pump (Grey Beard Niagra) was then affixed to the top of the central chamber to circulate buffer across the tissue during clearing by pulling buffer from the temperature-controlled bath. The chamber and output from the pump were lowered into a bath of 37 °C SDS. Direct electrical current was then applied across the tissue to initiate clearing. After clearing, the tissue was taken out of the clearing chamber and washed in 0.1 M PBS for at least 12 h.

### Clearing temperature measurements

Tissue was left to incubate in SDS-clearing solution for 2 days at 37 °C, then allowed to cool to room temperature for at least 2 h. Tissue was then subjected to either MHD-accelerated or electrophoretic clearing (n = 6) for 30 min with four different voltages applied across the tissue (30, 40, 50, and 60 VDC) in a 37 °C SDS bath. After clearing the tissue was rapidly removed from the device and imaged with an infrared thermal imaging camera (Hti-Xintai: HT-18) on a room temperature background. The highest observed temperature from each sample was recorded and the tissue was allowed to cool down to room temperature prior to additional experiments at different voltages.

### Refractive index matching and light sheet microscopy

The tissue was transferred to “Optiview”^23^ refractive index matching solution and incubated at 37 °C for at least 12 h to achieve optical clarity through RI matching (Fig. 5A^23^). Samples were imaged at 5X or 20X magnification with a lightsheet microscope adapted for a 1.45 RI imaging solution (Zeiss Z1).

### Measures of clearing efficacy

Tissue was left to incubate in SDS-clearing solution for 2 days at 37 °C. Tissue was then subjected to either MHD-accelerated or electrophoretic clearing (n = 6) for 24 h. Clearing was interrupted at 0 h, 6 h, 12 h, and 24 h. Tissue was washed with 0.01 M PBS overnight, then equilibrated to RI 1.43 in Optiview^23^ for at least two days at 37 °C. Tissue transparency was then measured by the percentage of light transmitted through the tissue suspended in an Optiview solution^23^. Light transmission was measured using a wide-spectrum light-source and calibrated photodiode. The sample was then washed in 0.01 M PBS overnight, then equilibrated to SDS-clearing solution for 2 days at 37 °C before clearing continued up to 24 h per sample. Data across all samples at each time were fit with a saturating exponential curve in MATLAB.

### MHD-accelerated staining of fixed tissue with methylene blue

Penetration of methylene blue into a 1 cm^3^ cube of homogeneous brain tissue under MHD force was tested over 1, 2, and 4 h (N = 1). Cubes of uncleared sheep brain tissue were equilibrated to the antibody labeling buffer solution for 12 h. The tissue was then placed at the intersection of a strong magnetic and electric field (30 V DC) and submerged in a solution of methylene blue (0.1 M) buffered to pH 9.5 (37 °C). The orientation of the electric field was reversed at 15-min intervals for 3 min. Three samples were labeled using this approach for 1, 2 or 4 h. Following the stain, the tissue was bisected and imaged. A control sample was incubated in the same solution (37 °C) for 4 h without the application of any active force. This sample was bisected and imaged as the others.

### Comparative staining of methylene blue into agarose cubes as a result of various strengths of electrical force conjugated to MHD force

15 1 cm^3^ of 3% agarose were subjected to labeling methylene blue labeling by MHD force for 0, 5, 10, 15, 30, 60, or 120 min at varied electrical field strengths. The distance penetrated into the agarose cubes was measure after bisection and plotted against staining time with 10, 20, or 30 V in a constant magnetic field.

### Antibody labeling

Delipidated tissue was placed inside of a 2-inch length of 0.25-inch diameter dialysis tubing (6–8 kDa); Spectrum). After equilibration, samples were incubated in an antibody solution inside dialysis tubing at the center of intersecting electrical and magnetic fields where the MHD force was strongest (Fig. 6). Confining the tissue sample inside dialysis tubing reduced the volume of antibody required for labeling and protected the tissue sample and antibody solution from direct exposure to the electrodes. Magnets (Applied magnets; NB057-6-N52) were placed on the top and bottom of the MHD labeling device creating a central chamber Fig. 6B). The ends of the dialysis tubing were connected to 9.5 mm diameter vinyl tubing (ThermoFisher: S504591) using 0.25-inch Leur lock barbs (Cole-Parmer; UX-45501-20) to create a torus-shaped chamber allowing the antibody solution to circulate continuously and provide an even and continuous source of antibody to the tissue sample (Fig. 6). Antibody solution (4.5 mL; 0.1 M borate buffer titrated to pH 9.5 with 0.1 M LiOH, 1% heparin, 0.1% Triton X-100; 1:500 primary antibody) was transferred into the dialysis tubing using a 5 mL syringe. The labeling chamber was submerged in a 1L tub containing electrophoresis solution (0.1 M Borate Buffer pH 9.5/0.1% Triton X-100 solution). A 5 mL syringe filled with the buffer solution was attached to the circulation line to maintain constant pressure inside of the dialysis tube. 60 V DC (~ 0.2 Amps) was applied across the electrodes for 15 min, followed by 3 min of inactivity repeatedly for 12 h to drive antibodies into the tissue sample. The system was held at 37 °C and protected from ambient light to minimize bleaching of fluorophores throughout the procedure. A list of antibodies successfully used for CLARITY tissue samples can be found at: http://wiki.claritytechniques.org/index.php/Immunostaining#Primary_antibodies_used_in_CLARITY_literature.

Following each round of MHD-accelerated labeling, the antibody solution was replaced with a wash solution (0.1 M borate buffer titrated to pH 9.5 with 0.1 M LiOH, 1% heparin, 0.1% Triton X-100) and the tissue was exposed to 6-h of “active washing” using the same voltage settings. Labeled tissue was then washed in 0.01 M PBS for at least 12 h.

Colocalization analysis of labeling was performed using the ‘colocalize’ function in FIJI^24^ to calculate the Pearson’s coefficient of the correlation for pixel-randomized correlation. The figures presented represent z-projections (20 to 150 µm depth).

### Traditional immunohistochemistry

Mouse brains were dissected from highly anesthetized mice. These tissues were incubated in 4% PFA suspended in 0.01 M PBS at 4 °C. Tissue was sliced to 100 µm thickness on a vibratome and transferred to 0.01 M PBS or the electrophoresis buffer used in MHD-accelerated labeling. Slices were blocked in 10% FBS in 0.5% TritonX-100/PBS or electrophoresis buffer at room temperature for 1 h, then incubated in a 1:200 dilution of antibody in 10% FBS/PBS or electrophoresis buffer at room temperature for 2 h. The tissue was then washed three times in 0.05% TritonX-100/PBS or electrophoresis buffer for 30 min at room temperature.

## Supplementary Information

Supplementary Information 1.

Supplementary Information 2.

Supplementary Video 1.

Supplementary Video 2.

## Data Availability

All primary data is available upon request to the corresponding author.
