# Supplementary material for: Accelerated clearing and molecular labeling of biological tissues using magnetohydrodynamic force
Source: Sci Rep. 2021 Aug 12;11:16462. doi: 10.1038/s41598-021-95692-2 (PMC8360944; doi:10.1038/s41598-021-95692-2)
Supplement: Supplementary file 2 — Supplementary Information 2. [file 41598_2021_95692_MOESM2_ESM.docx]

**Figure S1:** Images of a single brain split along the sagittal plane with one hemisphere cleared with electric-only clearing A) and the other hemisphere cleared with MHD-accelerated clearing B).

**Figure S2:** Images of hypothalamus neurons expressing tdTomato under the control of the AVP-promoter. A) Cell population in a brain cleared using MHD-accelerated labeling (top), and higher magnification of the dashed box in the top image (bottom). B) Cell population in a brain cleared using electric-only force (top), and higher magnification of the dashed box in the top image (bottom).

**Figure S3:** A) Penetration of methylene blue into a 1 cm^3^ cube of homogeneous brain tissue as a result of MHD force over 1, 2, and 4 hours (N = 1). The fourth image shows a comparative 4-hour stain without MHD force. The arrows on the left-hand side of the images demonstrate the direction of the MHD force with respect to the tissue. The length of the arrows demonstrates the proportion of time when the MHD force was aimed in the direction indicated by each arrow. B) shows the comparative staining of methylene blue into agarose cubes as a result of various strengths of electrical force conjugated to MHD force. The distance the methylene blue penetrated into the agarose cubes is measured against the amount of time stained with 10, 20, or 30V conjugated to a constant magnetic field. ​

**Figure S4:** Antibody labeling of a 100 µm, PFA-fixed slice from a mouse that expresses tdTomato under control of the AVP promoter (red; AVP-cre X rosa26-lsl-tdTomato) with α-vassopressin antibody (green) using a pH 7.0, PBS-based buffer A) or the pH 9.5 electrophoresis buffer used in MHD-accelerated labeling. In both A) and B) endogenous fluorescence is on the left, antibody fluorescence is on the right and a merge of the two images is in the center.


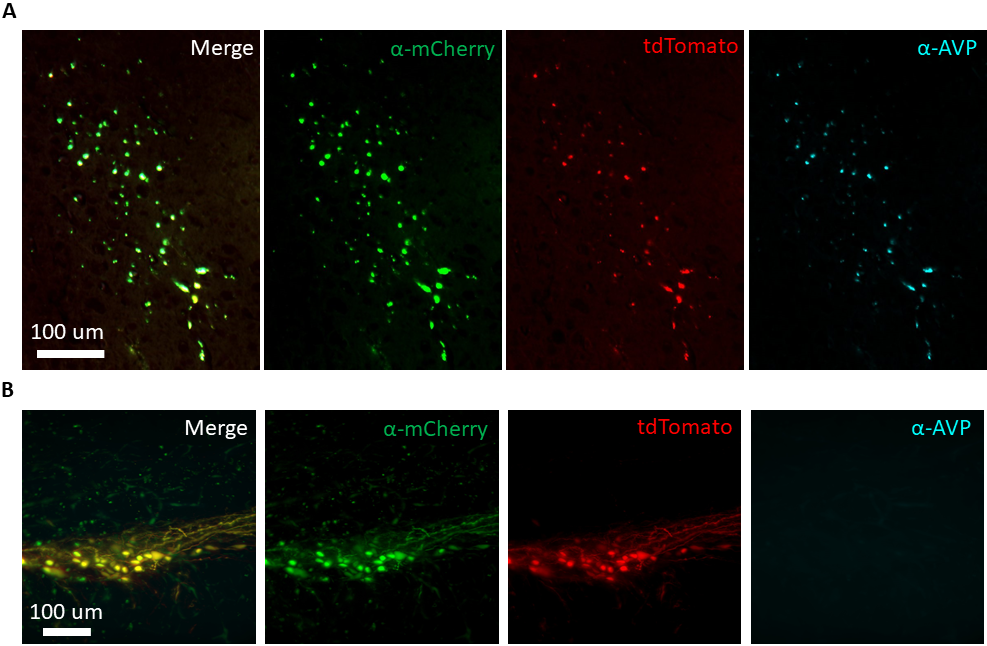


**Figure S5**: **MHD-accelerated antibody labeling of mouse tissue with multiple antibody probes.** A) Cells that express tdTomato (red) under the control of the AVP-promoter labeled with antibodies targeting mCherry (green) and AVP (cyan). Images from left to right show merged channels, α-mCherry antibody with Alexafluor 488 nm secondary antibody, tdTomato (Ai9) crossed with the AVP-Cre transgenic mice, and α-AVP with Alexafluor 647 nm secondary antibody (Pearson’s coefficient α-mCherry to tdTomato: r = 0.67; Pearson’s coefficient α-AVP to tdTomato: r = 0.64). B) tdTomato-expressing cells (red) in the mitral and tufted layer of the main olfactory bulb labeled with the same antibodies targeting mCherry (green) and AVP (cyan; panel order the same as in A). This MOB population of neurons consistently expresses tdTomato in the adult brain (likely due to developmentally restricted expression of AVP) but does not express AVP in the adult (Pearson’s coefficient α-mCherry to tdTomato: r = 0.86; Pearson’s coefficient α-AVP to tdTomato: r = 0.54).

**Solutions**

- Hydrogel monomer solution: 4% acrylamide (Sigma Aldrich; A3553); 0.05% bis acrylamide (Bio Rad; 1610142); 0.25% Initiator (Fisher Scientific; NC0632395); 0.01 M PBS (Fisher Scientific; BP2944100)
- Clearing Solution: 4% sodium dodecyl sulfate in 200 mM Boric Acid brought to pH 8.5 with 0.1 M NaOH
- Electrophoresis Buffer: 0.1 M Borate Buffer and 0.1% Triton X-100 (Fisher Scientific; 85111) brought to pH 9.5 with 0.1 M LiOH
- Optiview: Sodium Diatrizoate: 0.173 M (Sigma Alrdrich; **S4506**), Meglumine Diatrizoate: 0.816 M (Sigma Aldrich; **M5266),** Diatrizoic Acid 0.816 M (Chem Impex; 24150), pH 8 EDTA: 0.00005 M, Tween-20: $9\times{10}^{-7}M$ (Sigma Aldrich; **P1379)**

**MHD-accelerated tissue clearing protocol**

1. Incubate sample of PFA-perfused tissue in hydrogel monomer solution overnight at 4˚ C.
   - Hydrogel: (4% acrylamide (Sigma Aldrich; A3553); 0.05% bis acrylamide (Bio Rad; 1610142); 0.25% Initiator (Fisher Scientific; NC0632395); 0.01 M PBS (Fisher Scientific; BP2944100)).
2. Degas hydrogel with sample in it with one liter of nitrogen gas
3. Polymerize sample by placing in a water bath at 37 ˚ C for 2 – 3 hours
4. Remove excess hydrogel from the surface of the sample
5. Place in sample in 40 mL clearing solution (4% SDS solution in 200 mM Boric Acid) at 37 ˚ C for 2 days
6. Transfer sample to the basket in the central channel of the MHD-accelerated clearing device and submerge in a five-liter bath of clearing solution
7. Apply 0.3 to 0.5 Amps across the tissue at 36.7 ˚ C (we find 0.3 Amps for 12 hours to be an effective starting point).

**Refractive Index-Matching**

1. Wash tissue sample in 0.01 PBS at 37 ˚ C overnight or for 8 to 12 hours
2. Incubate tissue in 30 mL Optiview for two days

**MHD-accelerated antibody labeling protocol**

Preparation

1. Cut dialysis (6-8 kDa Spectra Por1) tubing to 2.25” + size of tissue sample (along the longest axis)
2. Equilibrate dialysis tubing in dH_2_O for at least 30 min at RT in bath of electrophoresis buffer
3. Fill dialysis tubing and one-liter chamber with Electrophoresis Buffer
   1. Electrophoresis Buffer: 0.1 M Borate Buffer brought to pH 9.5 with 0.1 M LiOH and 0.1% Triton X-100
4. Place tissue in the center of the dialysis tubing
5. Submerge dialysis tubing with tissue inside in one-liter chamber filled with Electrophoresis Buffer
6. Prepare a concentrated 300 µL solution of antibody in electrophoresis buffer
   1. 1:30 antibody, 1% Heparin
7. Load antibody solution into a syringe

Device setup

1. Submerge the torus-shaped tube in electrophoresis buffer
   1. Ensure that all air in the tubing is replaced with electrophoresis buffer
2. Attach each end of the dialysis tubing, with the tissue inside, to the corresponding ends of the torus-shaped tubing
   1. Run the dialysis tubing through the two openings on either side of the acrylic device
   2. Fit the ends of the dialysis tubing over the barbs on the ends of the torus-shaped tubing
   3. Use nylon screws to tighten an acrylic plate onto the end of the barbs on the outer edges of the acrylic device, pushing them into the angled fittings
3. Ensure that the entire tubing system is water-tight with no leaks and no air bubbles

Active antibody incubation:

1. Place the device, with the tube intact, over a waterproofed N52 neodymium magnet and affix the electrode array over the device to create a channel that is held in place, with an electrode on either side of the tissue, by the attraction between the top and bottom magnets
2. Submerge the intact device in electrophoresis buffer in the one-liter chamber
3. Use the inputs to the torus-shaped tube to flush the concentrated antibody solution in the syringe into the intact torus and dialysis tube system
4. Ensure that there are no bubbles or leaks in the system and that the tissue is positioned in the center of the dialysis tubing at the intersection of the magnetic and electric fields
5. Provide power to the electrode array by activating the power supply at 30-60 VDC and 0.2 to 0.3 Amps
6. Every two hours turn off power to the electrodes and use the torus tube input to flush 300 µL electrophoresis buffer into the system for the entirety of the labeling session (12 hours)

Active wash:

1. Flush the antibody solution out of the system and refill with pure electrophoresis solution
2. Repeat the ‘active antibody incubation’ protocol without additional antibody for an additional 6 to 12 hours

**Antibodies used:**

Zebrafish:

Primary: Mouse IgG anti-Acetylated Tubulin antibody (Sigma-Aldrich)

Secondary: Goat IgG anti-mouse Alexa 647 nm (ThermoFisher)

Nudibranch:

Primary: Rabbit anti-5-HT (Immunostar)

Secondary: Goat anti-rabbit Alexa 488 nm (ThermoFisher)

Mouse:

Primary 1: Rabbit anti-oxytocin antibody (Immunostar)

Primary 2: Rabbit anti-AVP antibody (AbCam)

Primary 3: Rat anti-mCherry (ThermoFisher)

Secondary 1: Goat IgG anti-rabbit Alexa 647 nm (Jackson ImmunoResearch)

Secondary 2: Goat IgG anti-rabbit Alexa 488 nm (ThermoFisher)

Secondary 3: Donkey IgG anti-rat Alexa 488 nm (ThermoFisher)

**Materials used to build clearing device:**

0.25 mm diameter 99.9% platinum wire (Sigma-Aldrich; 349402)

Silicone adhesive (Grainger; 4UH03)

1” x 2” x 1” N52 Neodymium magnet (Applied magnets; NB057-6-N52)

1/8” thickness acrylic sheet (Delvie’s Plastics)

Nylon bolts (Mcmaster-Carr; 93939A734)

**Materials used to build antibody labeling device:**

1” x 2” x 0.5” N52 Neodymium Magnet (Applied magnets; NB057-6-N52)

0.25 mm diameter 99.9% platinum wire (Sigma-Aldrich; 349402)

1” thickness acrylic sheet (Delvie’s Plastics)

1/8” thickness acrylic sheet (Delvie’s Plastics)

0.25” diameter Spectra Por 1 6-8 kDa dialysis tubing (Spectra Por; 132645)

0.5” cubed N52 Neodymium Magnet (Applied magnets; NB022-N52)

1/8” inner diameter vinyl tubing (ThermoFisher: S504591)

1/4” to 1/8” male-to-male tubing adapters (Cole-Parmer; UX-45501-20)

Nylon bolts (Mcmaster-Carr; 93939A734)

25-gauge winged infusion needle and IV (Fisher Scientific; 22-289913)
